# Endoplasmic Reticulum Stress Signaling as a Therapeutic Target in Malignant Pleural Mesothelioma

**DOI:** 10.3390/cancers11101502

**Published:** 2019-10-08

**Authors:** Duo Xu, Haitang Yang, Zhang Yang, Sabina Berezowska, Yanyun Gao, Shun-Qing Liang, Thomas M. Marti, Sean R. R. Hall, Patrick Dorn, Gregor J. Kocher, Ralph A. Schmid, Ren-Wang Peng

**Affiliations:** 1 Department of General Thoracic Surgery, Department for BioMedical Research (DBMR), Inselspital, Bern University Hospital, University of Bern, 3008 Bern, Switzerland; duo.xu@dbmr.unibe.ch (D.X.); haitang.yang@dbmr.unibe.ch (H.Y.); zhang.yang@dbmr.unibe.ch (Z.Y.); yanyun.gao@dbmr.unibe.ch (Y.G.); shun-qing.liang@umassmed.edu (S.-Q.L.); Thomas.Marti@insel.ch (T.M.M.); Sean.Hall@insel.ch (S.R.R.H.); Patrick.Dorn@insel.ch (P.D.); Gregor.Kocher@insel.ch (G.J.K.); 2 Graduate School for Cellular and Biomedical Sciences, University of Bern, 3008 Bern, Switzerland; 3 Institute of Pathology, University of Bern, 3008 Bern, Switzerland; sabina.berezowska@pathology.unibe.ch

**Keywords:** malignant pleural mesothelioma (MPM), endoplasmic reticulum (ER) stress, unfolded protein response (UPR), HA15, autophagy

## Abstract

Malignant pleural mesothelioma (MPM) is a lethal cancer with limited treatment options. No targeted therapy has emerged yet. Here, we performed an integrated molecular characterization of patient tumors in the TCGA dataset, and discovered that endoplasmic reticulum (ER) stress and the adaptive unfolded protein response (UPR) signaling are characteristically deregulated in MPM. Consequently, pharmacological perturbation of ER stress/UPR axis by HA15, an agent that induces persistent proteotoxic stress in the ER, selectively suppresses the viability of MPM cells including those refractory to standard chemotherapy. Mechanically, HA15 augments the already high basal level of ER stress in MPM cells, embarks pro-apoptotic malfunctional UPR and autophagy, which eventually induces cell death in MPM. Importantly, HA15 exerts anti-MPM effectiveness in a mouse model of patient-derived xenografts (PDX) without eliciting overt toxicity when compared to chemotherapy. Our results revealed that programs orchestrating ER stress/UPR signaling represent therapeutic vulnerabilities in MPM and validate HA15 as a promising agent to treat patients with MPM, naïve or resistant to chemotherapy.

## 1. Introduction

Malignant pleural mesothelioma (MPM) is a rare but aggressive cancer [1,2], consisting of epithelioid, mixed (biphasic) and sarcomatoid histological subtypes [3]. Despite progress in the understanding of the disease etiology, clinical management of MPM patients remains a significant but unmet challenge [4]. Whereas aggressive surgery is amenable for early-stage tumors [5], a majority (80%) of patients with MPM are diagnosed at advanced stages, for which a dual chemotherapy regimen that combines cisplatin and pemetrexed is the only clinically approved therapy [6]. However, this systemic treatment only mildly improves patient survival (by three months only), as drug resistance, de novo, and/or acquired after the treatment, prevails [7]. 

Comprehensive genomic studies revealed that MPM is predominantly driven by loss of function mutations in tumor suppressor genes, most commonly the cyclin-dependent kinase inhibitor 2A/2B gene (*CDKN2A/2B*), BRCA1 associated protein 1 gene (*BAP1*), neurofibromin 2 gene (*NF2*), whereas therapeutically tractable oncogenic drivers are rare [8,9,10]. The lack of druggable activating mutations [9,11,12] have significantly hampered the development of targeted therapies for MPM, which, however, suggests the importance of identifying and targeting functional cancer dependencies rather than specific driver mutations to combat MPM. 

Cancer cells evolve in response to changes in oncogenic signaling and environmental pressures, for example, exacerbated secretory capacity, genomic instability and hypoxia [13,14]. Many stress-responsive mechanisms converge at an anabolic switch that increases protein metabolism, which induces proteotoxic endoplasmic reticulum (ER) stress, and in turn invokes the unfolded protein response (UPR) [15,16,17]. The UPR is mediated by three major signaling cascades initiated by so-called ER stress sensors: double-stranded RNA-activated protein kinase (PKR)-like ER kinase (PERK), inositol-requiring enzyme 1α (IRE1α), and activating transcription factor 6 (ATF6). PERK, IRE1α, and ATF6 are ER membrane proteins that, at the steady state, are complexed with the chaperone protein glucose-regulated protein 78 (GRP78, also known as BiP). When threatened by increased protein-folding demand (ER stress), BiP is released, which activates PERK, IRE1α, and ATF6, and in turn, their downstream effectors to alleviate proteotoxic stress placed on the ER and to restore ER homoeostasis [18]. However, if the stress is irresolvable, the UPR signaling will trigger apoptosis/autophagy, leading to cell death [19,20].

UPR dysfunction is implicated in many human diseases, for example, neurodegenerative, cardiovascular, and metabolic disorders. Hyperactive UPR has been reported in Alzheimer’s disease (AD), progressive supranuclear palsy, and familial FTD (frontotemporal degeneration) with parkinsonism linked to chromosome 17 [21]. Recent studies have also uncovered that chronic ER stress is linked with endothelial dysfunction in cardiovascular diseases by increasing oxidative stress [22], and with insulin resistance and increased lipogenesis involved in metabolic disorders such as type II diabetes and obesity [23]. Deregulation of ER stress/UPR is also not uncommon in cancers [24,25] and many rely on the stress response signaling for survival or evasive resistance to cytotoxic therapeutics [26]. As such, targeting the UPR has emerged as a promising strategy for cancer therapy [27,28]. 

A recent study by Cerezo et al., identified a new ER stress amplifier, termed HA15. HA15 binds to and inhibits the ATPase activity of BiP, thereby dissociating BiP from PERK, IRE1α, and ATF6 and triggering ER stress [29]. The anti-tumor effects of HA15 has been demonstrated in a variety of human cancers including melanoma, breast, pancreas, and adrenocortical carcinoma [29,30]. In this study, we showed that deregulation of ER stress/UPR signaling is a hallmark of MPM, which confers a specific vulnerability of therapeutic potential. Consequently, MPM cells, regardless of chemo-naïve or -resistant, are particularly susceptible to HA15. We further demonstrated that the antitumor effect of HA15 is attributable to its ability to induce excessive proteotoxic stress in the ER, which initiates malfunctional UPR, autophagy, and eventually the onset of apoptosis.

## 2. Results

### 2.1. ER Stress and the Adaptive UPR Is Deregulated in Patients’ MPM 

**Figure 1:**
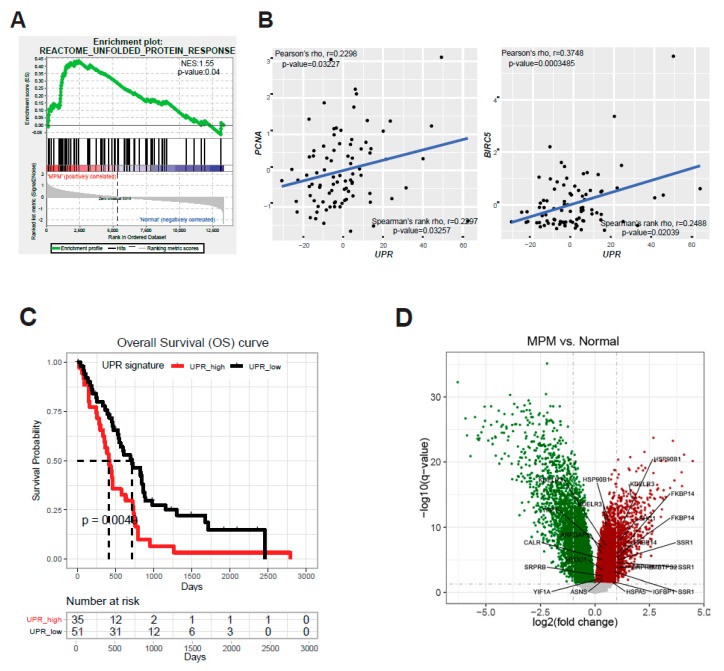

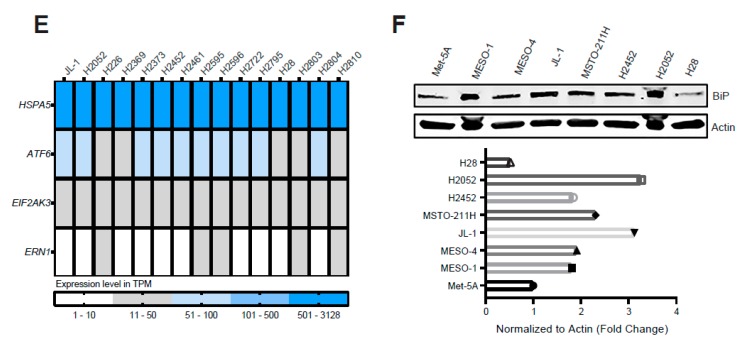
Endoplasmic reticulum (ER) stress/unfolded protein response (UPR) signaling is deregulated in malignant pleural mesothelioma (MPM). (**A**) Gene set enrichment analysis (GSEA) of the dataset (GSE2549) revealed significant enrichment of UPR in MPM tumor samples. (**B**) The UPR gene signature was positively correlated with the proliferative marker *PCNA* and anti-apoptotic marker SURVIVIN/*BIRC5*. Gene expression data of MPM were downloaded from TCGA, with Pearson/Spearman coefficients and *p*-value determined using R (version 3.4.3). (**C**) Kaplan-Meier analysis of a TCGA cohort of MPM patients (*n* = 86). The UPR gene signature was dichotomized based on the optimal cut-off value, and patient survival data were extracted for further analysis using R (Version 3.4.3). The *p*-value was calculated by the log-rank test. (**D**) The volcano plot of transcriptional comparison between the patients’ MPM vs. normal lung tissues (GSE51024). (**E**) Heatmap of key UPR gene expression (TPM) in the MPM cell lines. The expression profile was obtained from the European Bioinformatics Institute of European Molecular Biology Laboratory (EMBL-EBI). (**F**) Immunoblots of BiP in normal mesothelial cells (Met-5A) and MPM cell lines. Relative expression of BiP is shown underneath, with signal intensity quantified by ImageJ and normalized to the loading control (β-actin). The value in the Met-5A was set as 1.

To unravel cellular pathways that may represent specific dependencies of MPM, we performed an integrated analysis of patients’ MPM based on transcriptomic and clinical data available at the Cancer Genome Atlas (TCGA) [31]. Gene enrichment set analysis (GESA) revealed that genes involved in ER stress and the adaptive UPR (UPR gene signature) were significantly enriched in the patients’ tumors compared to normal lung/pleura tissues (Figure 1A). Consistently, the UPR gene signature was positively correlated with proliferation and resistance to apoptosis, assessed by the expression of *PCNA* and *BIRC5*, encoding the proliferative marker proliferating cell nuclear antigen (PCNA) and anti-apoptotic protein SURVIVIN (also called baculoviral inhibitor of apoptosis repeat-containing 5 or BIRC5), respectively (Figure 1B). Notably, the UPR gene signature is of predictive value for patients with MPM, with an elevated UPR signature associated with poor prognosis (Figure 1C). Further examination of the transcriptomic data of patient-derived MPM [32] revealed that numerous genes involved in ER-related functions and/or proteostatic processes (i.e., *FKBP14* [peptidyl-prolyl *cis*-trans isomerase (PPIase) FKBP14], *SSR1* (translocon-associated protein subunit alpha or TRAPα), *HSP90B1* (heat shock protein 90 beta family member 1) and *HSPA5/GRP78* (BiP)) are expressed at high levels in MPM compared to normal tissues (Figure 1D). Of particular interest, *HSPA5/GRP78* and its protein product BiP, a key ER chaperone and a master regulator of ER stress and the adaptive UPR, were generally overexpressed in MPM cells, except for H28, which had a lower BiP protein level than Met-5A (Figure 1E,F). Thus, deregulation of ER stress/UPR signaling is a characteristic feature for MPM and associated with poor prognosis in MPM patients. The original pictures of Western blotting can be found in Figure S1.

### 2.2. Excessive ER Stress Induced by HA15 Selectively Impairs MPM Cells 

**Figure 2:**
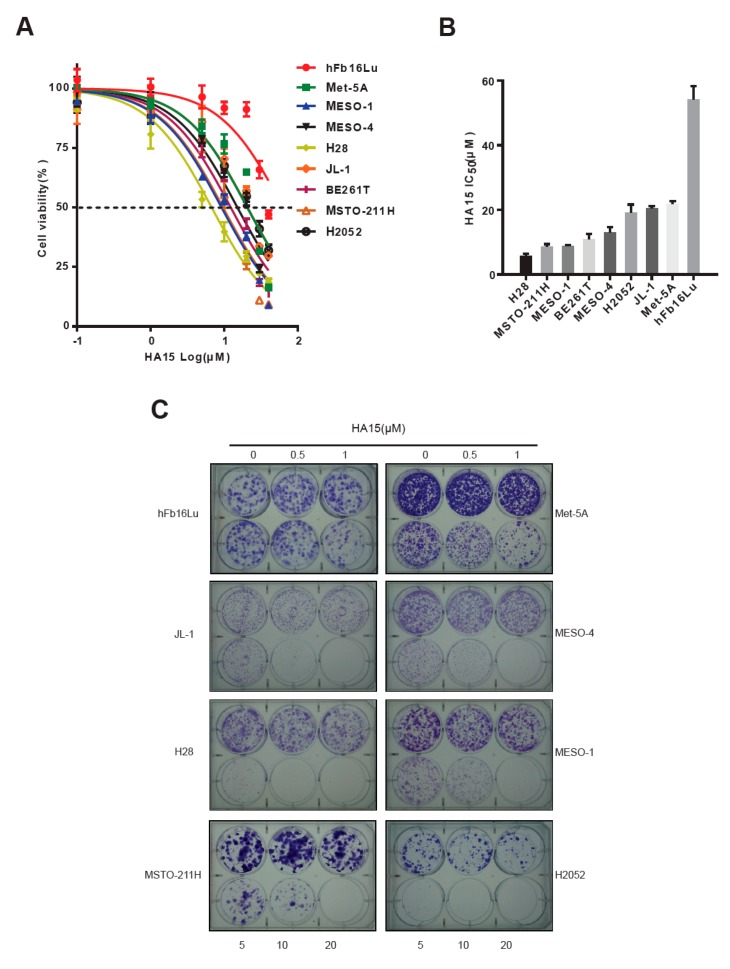

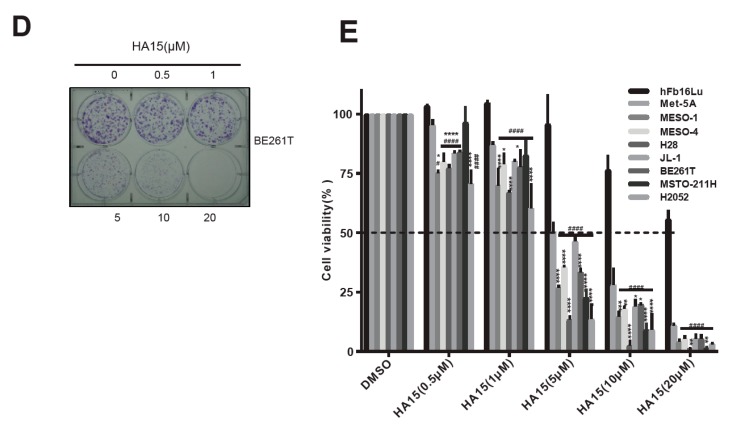
Excess ER stress induced by HA15 selectively inhibits MPM cells. (**A**,**B**) Cell viability and IC_50_ of normal human lung fibroblasts (hFb16Lu), normal human mesothelial (Met-5A), and MPM cells after treatment with HA15 for 72 h. Data are presented as mean ± s.d. A representative result is presented (*n* = 2). (**C**–**E**) Clonogenic assay of MPM cell lines (**C**), primary MPM culture (BE261T) (**D**), normal human lung fibroblasts (hFb16Lu), and normal human mesothelial (Met-5A) after being treated with HA15. Colonies were stained and quantified (right panel) after 14 days. Representative images of three independent experiments (*n* = 3) are shown. Quantification (**E**) was based on the results of (**C**) and (**D**). Data are presented as mean ± s.d. (*n* = 3). ^#^ *p* < 0.05, ^####^ *p* < 0.0001 by two-way ANOVA with Dunnett’s multiple comparisons test, when compared with hFb16Lu. * *p* < 0.05, ** *p* < 0.01, *** *p* < 0.005, and **** *p* < 0.0001 by two-way ANOVA with Dunnett’s multiple comparisons test, when compared with Met-5A.

To address if ER stress/UPR signaling can be a potential therapeutic target in MPM, we treated a panel of cells including MPM cell lines across all histological subtypes (MESO-1, MESO-4, H28, JL-1, MSTO-211H, and H2052), a primary MPM culture (BE261T) and non-transformed normal cells (hFb16lu, Met-5A), with HA15, an agent inducing excess ER stress by specifically targeting BiP [29]. HA15 preferentially suppressed cell viability of MPM cells, with the 50% inhibitory concentration (IC_50_) ranging from 5.71 to 20.51 µM, whereas it only mildly affected Met-5A and hFb16lu cells (Figure 2A,B). Clonogenic assay confirmed that HA15 is far more deleterious for MPM cells than fibroblast hFb16lu and mesothelial Met-5A cells (Figure 2C–E). These results suggest that targeting BiP by HA15 preferentially inhibits MPM cells and is therefore a potential therapeutic strategy for MPM.

### 2.3. Activation of Malfunctional UPR and Autophagy Underpins HA15 Effectiveness in MPM Cells 

**Figure 3:**
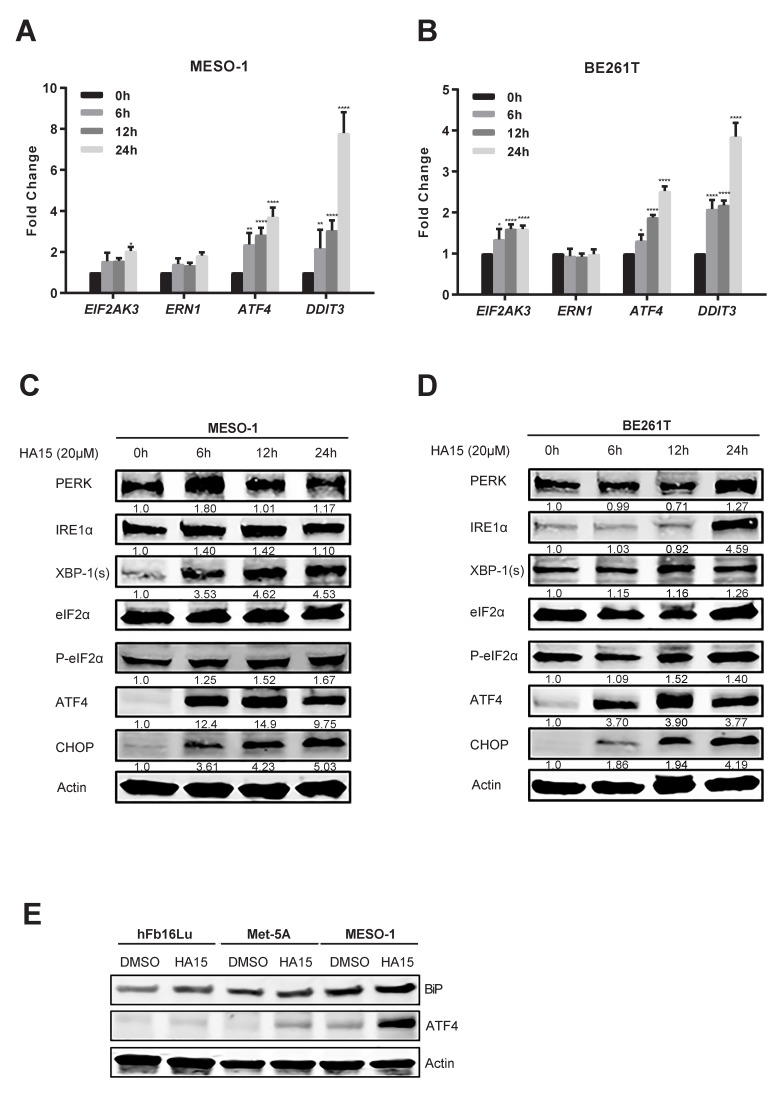
HA15-induced ER stress activates malfunctional UPR in MPM cells. (**A**,**B**) qRT-PCR of MESO-1 and BE261T cells after treatment with HA15 (10 uM) for the indicated time points. Data are presented as mean ± s.d. (*n* = 3). * *p* < 0.05, ** *p* < 0.01, and **** *p* < 0.0001 by two-way ANOVA with Dunnett’s multiple comparisons test. (**C**,**D**) Western blots of MESO-1 and BE261T cells after treatment with HA15 (20 uM) for the indicated time points. Quantification of the protein levels is shown under each band, with signal intensity measured by ImageJ and normalized to the loading control (β-actin). The value of the proteins in the vehicle group was set as 1. (**E**) Western blots of normal human lung fibroblasts (hFb16Lu), normal human mesothelial (Met-5A), and MPM cells (MESO-1) after treatment with HA15 (20 uM) for 24 h. Protein quantification is shown underneath, whereby signal intensity was assessed by ImageJ and normalized to the loading control (β-actin), with the value of the DMSO group set as 1.

**Figure 4:**
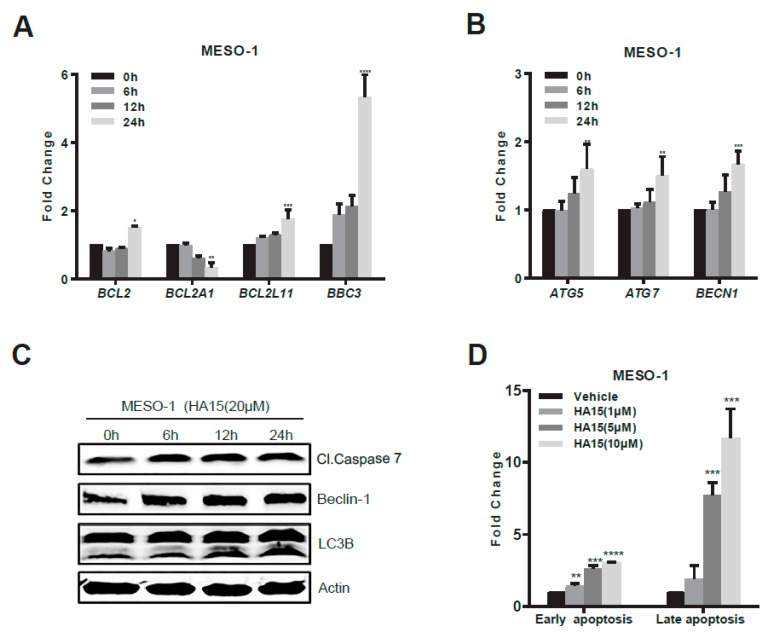

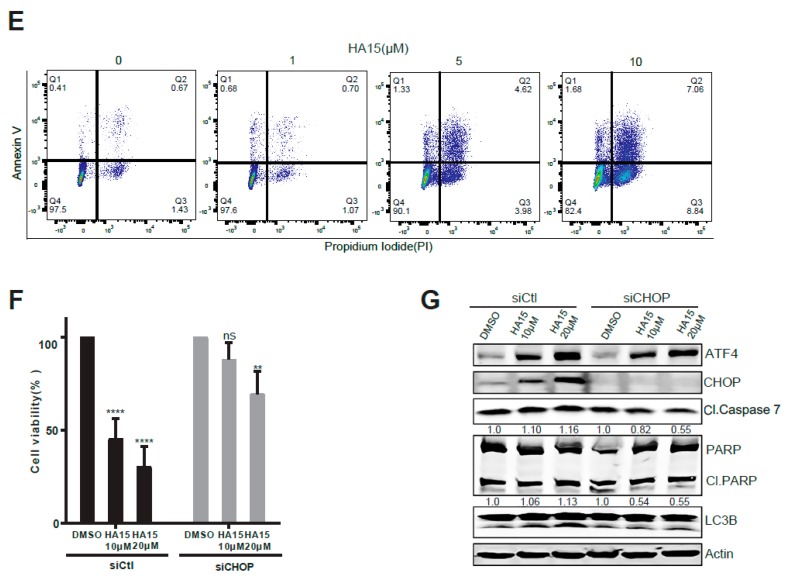
Activation of malfunctional UPR underpins HA15 efficacy in MPM cells. (**A**,**B**) qRT-PCR of MESO-1 cells after treated with HA15 (10 uM) for the indicated time points. Data are presented as mean ± s.d. (*n* = 3). * *p* < 0.05, ** *p* < 0.01, *** *p* < 0.005, and **** *p* < 0.0001 by two-way ANOVA with Dunnett’s multiple comparisons test. (**C**) Western blots of MESO-1 cells after treatment with HA15 (20 uM) for the indicated time points. Quantification of the protein levels is shown under each band, with signal intensity measured by ImageJ and normalized to the loading control (β-actin). The value of the proteins in the vehicle group was set as 1. (**D**,**E**) FACS-based apoptotic assay of MESO-1 cells after treatment with indicated doses of HA15 for 72 h. Data are presented as mean ± s.d. (*n* = 3). ** *p* < 0.01, *** *p* < 0.005, and **** *p* < 0.0001 by two-way ANOVA with Dunnett’s multiple comparisons test. A representative FACS plot is shown (**E**) with early and late apoptosis quantified by the Annexin V^+^/PI^-^ and Annexin V^+^/PI^+^ population, respectively. (**F**) MESO-1 cells transfected with the control (siCtrl) or CHOP siRNAs (siCHOP) were treated with indicated doses of HA15. Cell viability was measured 48 h after treatment (**F**). Data are presented as mean ± s.d. (*n* = 3). ** *p* < 0.01, and **** *p* < 0.0001 by two-way ANOVA with Dunnett’s multiple comparisons test. (**G**) Cell lysates were prepared from MESO-1 cells transfected with the control (siCtrl) or CHOP siRNAs (siCHOP) and analyzed by immunoblots. Protein quantification is shown underneath, whereby signal intensity was assessed by ImageJ and normalized to the loading control (β-actin), with the value of the DMSO group set as 1.

Next, we addressed the molecular underpinnings of HA15 effects in MPM. HA15 induced persistent ER stress in MPM cells, measured by increased expression of key UPR genes [*EIF2AK3* (PERK), *ERN1* (IRE1α), *ATF4* (ATF4), XBP-1s, *DDIT3* (CHOP)] at both mRNA and protein levels (Figure 3A–D). Notably, this HA15-induced increase of UPR gene expression (ATF4) did not occur in hFb16lu and Met-5A cells (Figure 3E). Moreover, HA15 treatment markedly augmented autophagic genes [*ATG5* (Autophagy protein 5), *ATG7* (Autophagy protein 7), *BECN1* (Beclin-1), and LC3B-II] (Figure 4A–C) and induced massive cell death in MESO-1 cells, marked by the time- and dose-dependent increase of pro-apoptotic markers [*BCL2L11* (BIM), *BBC3* (PUMA), and cleaved caspase 7 (Cl.Caspase 7)] (Figure 4A–C). Flow cytometry-based measurement of apoptotic cell populations confirmed that the HA15 treatment resulted in MPM cell death in a dose-dependent manner (Figure 4D,E). 

To test if the ER stress/UPR signaling axis is functionally important for HA15 effects on MPM cells, we knocked down *DDIT3* (encoding CHOP), a key effector of malfunctional UPR that leads to apoptosis. While HA15 dose-dependently decreased cell viability and concomitantly increased CHOP, apoptosis (cleaved caspase 7/PARP), and autophagy (LC3B-II/LC3B-I ratio) in MESO-1 cells with intact CHOP (siControl), CHOP depletion significantly attenuated HA15-induced growth inhibition, apoptotic, and autophagic indices in MESO-1 cells (Figure 4F,G). As expected, ATF4, which acts upstream of CHOP, was largely unaffected by CHOP downregulation and remained active in response to HA15 (Figure 4G). These data delineate that HA15 selectively triggers MPM cell death by inducing excessive ER stress and the onset of malfunctional UPR and autophagy.

### 2.4. HA15 Potently Inhibits Chemo-Resistant MPM Cells 

**Figure 5:**
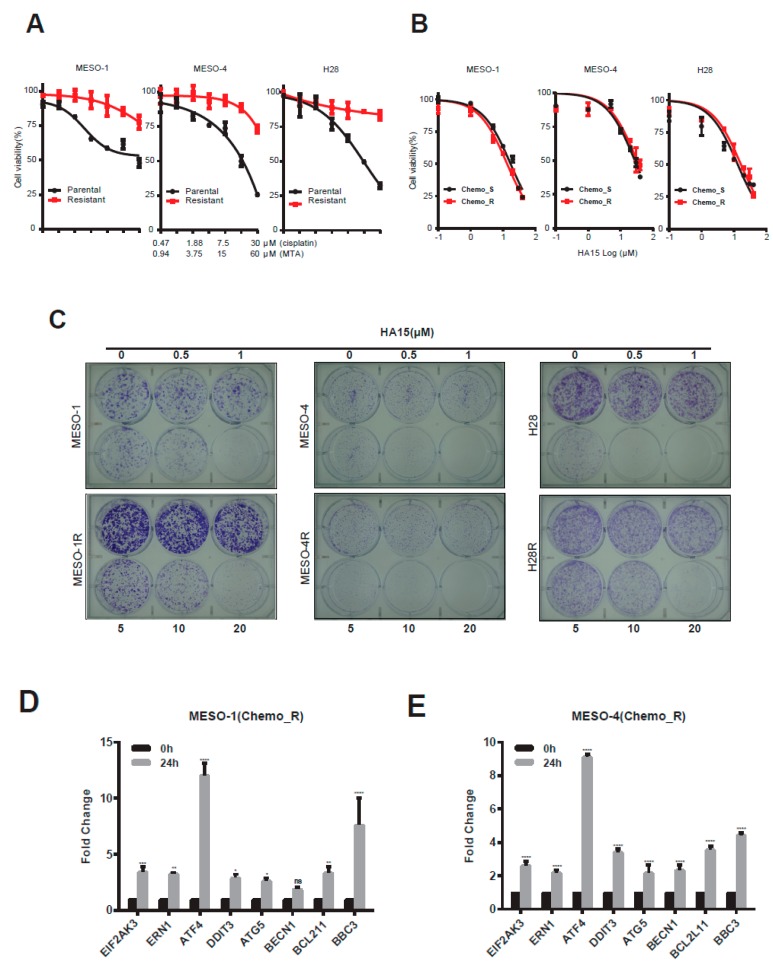

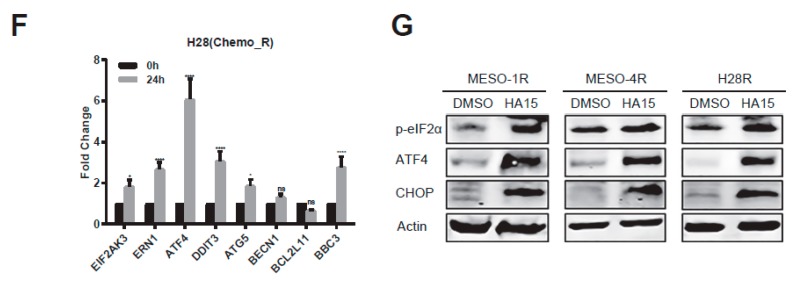
HA15 overcomes chemo-resistance in MPM cells. (**A**,**B**) Viability assay of parental (Chemo_S) and resistant (Chemo_R) MPM cells after treatment with cisplatin/pemetrexed or HA15 for 72 h. Data are presented as mean ± s.d. (**A**) representative result is presented (*n* = 2). (**C**) Clonogenic assay of parental MPM cells (MESO-1, MESO-4, H28) and resistant MPM cells (MESO-1R, MESO-4R, H28R) treated with HA15 for 14 days. Representative images of three independent experiments (*n* = 3) are shown. (**D**–**F**) qRT-PCR of resistant MPM cells after treated with HA15 (20 µM) for 24 h. Data are presented as mean ± s.d. (*n* = 3). * *p* < 0.05, ** *p* < 0.01, *** *p* < 0.005, and **** *p* < 0.0001 by two-way ANOVA with Sidak’s multiple comparisons test. (**G**) Immunoblots of chemo-resistant MPM cells treated with HA15 (20 µM) or vehicle (DMSO) for 24 h. Protein quantification is shown underneath, whereby signal intensity was assessed by ImageJ and normalized to the loading control (β-actin), with the value of the DMSO group set as 1.

To test if the effectiveness of HA15 can be extended to MPM cells resistant to standard chemotherapy, we generated chemo-resistant MPM cells by chronic exposure to stepwise-increasing doses of cisplatin/pemetrexed (Figure 5A). Both chemo-resistant (_R) and parental (_S) MPM cells responded to HA15 in a dose-dependent manner (Figure 5B,C). Coherent with the results from parental chemo-naïve MPM cells (Figure 3), HA15 induced excess ER stress, malfunctional UPR, and apoptotic cell death in chemo-resistant MPM cells, gauged by significant upregulation of UPR genes (*EIF2AK3*, *ERN1*, *ATF4*, *DDIT3*), autophagy (*ATG5*, *BECN1*), and pro-apoptosis [*BCL2L11* (BIM), *BBC3* (PUMA)] (Figure 5D–F). Western blot analysis confirmed that HA15 invoked ER stress-activated malfunctional UPR (increased p-eIF2α and CHOP) in chemo-resistant MPM cells (Figure 5G), which is known to lead to apoptosis. 

### 2.5. HA15 Suppresses MPM Tumor Growth In Vivo 

**Figure 6:**
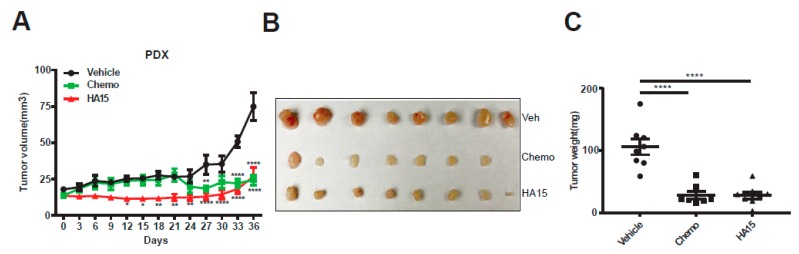

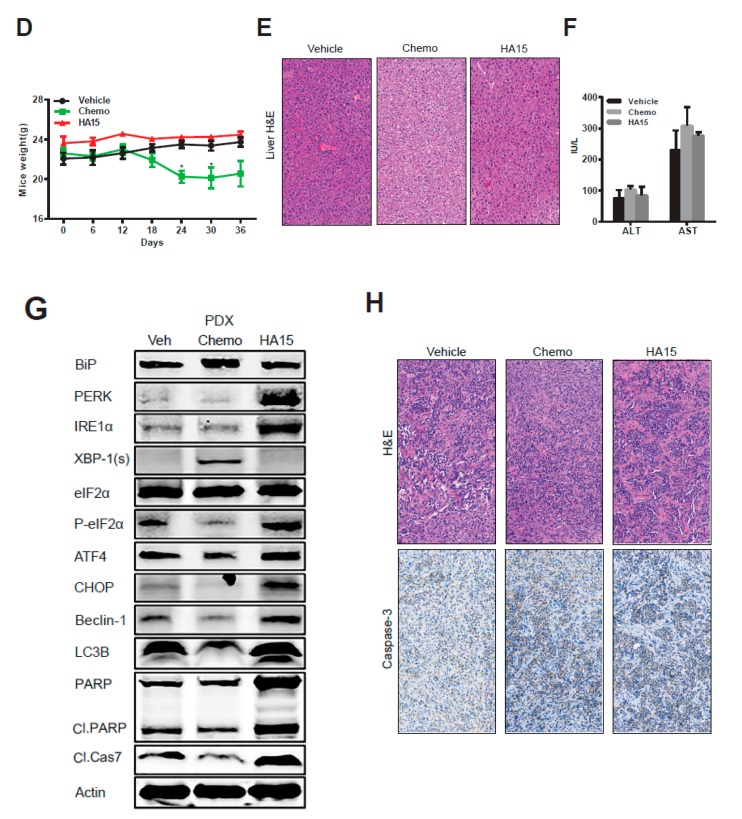
HA15 suppresses MPM tumor growth in vivo. (**A**) Growth curves of patient (BE261T)-derived xenograft (PDX) tumors treated with the vehicle, cisplatin/pemetrexed (3.75/83 mg/kg; chemo), or HA15 (0.7 mg/mouse) for the indicated time. Data are presented as mean ± SEM (*n* = 4). * *p* < 0.05, ** *p* < 0.01, and **** *p* < 0.0001 by two-way ANOVA with Dunnett’s multiple comparisons test. (**B**,**C**) Tumor size (**B**) and weights (**C**) of PDX (BE261T) tumors after the treatment. Data are presented as mean ± SEM (*n* = 4). * *p* < 0.05, ** *p* < 0.01, *** *p* < 0.001, and **** *p* < 0.0001 by one-way ANOVA with Dunnett’s multiple comparisons test. (**D**) Mice body weights during treatment with HA15, chemo, or vehicle. Data are presented as mean ± SEM (*n* = 4). * *p* < 0.05 by two-way ANOVA with Dunnett’s multiple comparisons test. (**E**) Liver histology from treated mice. Original overall magnification, ×400. (**F**) Transaminase (AST/ALT) activities of treated mice. Data are presented as mean ± SEM (*n* = 3). (**G**) Immunoblots of PDX tumors after the treatment. Protein quantification is shown underneath, whereby signal intensity was assessed by ImageJ and normalized to the loading control (β-actin), with the value of the vehicle group set as 1. (**H**,**E**) Staining and IHC analysis for caspase-3 of PDX tumors after the treatment. Original overall magnification, ×400.

Finally, we tested in vivo efficacy of HA15 through a comparison with standard chemotherapy (cisplatin/pemetrexed (MTA) in a patient (BE261T)-derived xenograft (PDX) model of MPM. Whereas HA15 and cisplatin/MTA alone delayed PDX tumor growth compared to the vehicle treatment, HA15 at the administrated dose exhibited greater anti-tumor effect than chemo (Figure 6A–C). Notably, the efficacy of chemo was achieved at the cost of high toxicity, but HA15 at the used dose showed no obvious side effects, monitored by body weights (Figure 6D) and a toxicity analysis that assessed liver histology and hepatic transaminase (AST/ALT) activities in the treated mice (Figure 6E,F). Importantly and in line with our in vitro results, PDX tumors after HA15 treatment displayed persistent activation of ER stress-induced UPR (PERK, IRE1α, p-eIF2α, ATF4) and autophagy (Beclin-1, LC3B-II), which was deemed anti-survival (CHOP), and enhanced cell death (Cl PARP, Cl Cas 7, Cl Cas 3) (Figure 6G,H). Thus, pharmacological perturbation of ER stress/UPR signaling by HA15 shows strong anti-MPM efficacy by inducing tumor cell death, validating HA15 as a potential therapeutic for MPM.

## 3. Discussion

We previously reported that dysregulated ER stress/UPR signaling is an important mechanism underlying resistance to standard cisplatin/pemetrexed chemotherapy, and that further induction of persistent ER stress overcomes chemo-resistance [26]. In this study, we discovered that deregulation of ER stress/UPR signaling is a characteristic feature for MPM, which confers a therapeutic vulnerability that normal mesothelial counterparts lack. We further demonstrated that pharmacologic augmentation of ER stress by HA15 selectively targets MPM without apparent side effects in immune-deficient NSG mice, leading to apoptotic cell death of MPM cells in vitro and potent suppression of MPM tumor growth in preclinical mouse models. Mechanistically, HA15 induces excessive proteotoxic pressures above the already high levels of ER stress in MPM, which embarks malfunctional UPR, autophagy, and eventually activates programmed cell death. These results provide a therapeutic rationale by targeting ER stress/UPR signaling in MPM and validate HA15 as a potential therapeutic for MPM. 

Perturbation of ER stress/UPR can be achieved by pharmacological strategies, and small molecules that target key components of the UPR machinery (e.g., the enzymatic activity of PERK (kinase), IRE1α (RNAse) and the eukaryotic initiation factor eIF2α) have been investigated in preclinical studies [33]. Although pharmacological modulation of ER stress/UPR has demonstrated significant anti-tumor activity in a variety of cancer models, undesired on-target side effects of ER stress modulators have remained incompletely understood. For instance, chronic administration of PERK inhibitors has been reported to impair pancreatic β-cells [34,35]. Furthermore, ER stress/UPR plays a key role in immune surveillance [14]. As a consequence, deregulation of the IRE1α-XBP1 axis has been reported to account for dysfunctional dendritic cells (DCs) and neutrophils [36], and CHOP is critical for the immune inhibitory activity of tumor-infiltrating myeloid-derived suppressor cells (MDSCs) and of CD8^+^ T cells [37,38]. 

Predominantly driven by pharmacologically intractable genetic alterations in tumor suppressers [8,9,10], MPM remains the epitome of a lethal malignancy recalcitrant to targeted therapy efforts. By capitalizing on an integrative approach, here, we discovered that ER stress/UPR signaling is significantly deregulated in MPM compared to normal mesothelial cells. In particular, the molecular chaperon BiP, a member of the heat shock protein 70 (HSP70) family and a master regulator of ER stress response, is generally expressed at a high level in patients’ MPM and established MPM cell lines. BiP cycles between the membrane and lumen of the ER, which functions as a molecular switch that senses the magnitude of ER stress and sets the threshold for the onset of ER stress-responsive UPR [39]. As the outcome of the UPR is bi-directional, pro-survival for readily relievable ER stress or pro-apoptotic if persistent and incurable ER stress emanates, targeting ER stress signaling aimed at tipping the UPR from a pro-survival to a pro-apoptotic mechanism, the latter executed by malfunctional UPR, has emerged as a promising strategy of cancer therapy [26]. As proof of our finding, MPM cells, regardless of chemotherapy-naïve or -resistant, were equally susceptible to HA15, a small molecule that induces excess ER stress by specifically targeting BiP [29]. Indeed, the antitumor effect of HA15 is intimately linked with its ability to augment UPR, as genetic depletion of CHOP, a master regulator of apoptotic cell death elicited by malfunctional UPR, abrogates HA15 effectiveness in MPM.

In summary, it remains paramount to identify new and effective therapeutic approaches to improve the clinical outcome of MPM patients. Our finding that deregulation of ER stress and the adaptive UPR sensitizes MPM cells to agents that induce excess ER stress and alter the adaptive UPR ushers in a therapeutically actionable strategy for MPM and supports further clinical investigations of HA15 to treat patients with MPM, although the potential side effects of HA15 on immunity remains to be addressed.

## 4. Materials and Methods

### 4.1. Cell Culture and Reagents

Normal human lung fibroblasts hFb16Lu (CCD-16Lu, RRID: CVCL_2378), normal human mesothelial cells Met-5A (MeT-5A, RRID: CVCL_3749), MPM cell lines H28 (NCI-H28, RRID: CVCL_1555), H2452 (NCI-H2452, RRID: CVCL_1553), and H2052 (NCI-H2052, RRID: CVCL_1518) were obtained from ATCC (American Type Culture Collection, Manassas, VA, USA). MPM cell lines MESO-1 (ACC-MESO-1, RRID: CVCL_5113) and MESO-4 (ACC-MESO-4, RRID: CVCL_5114) were obtained from RIKEN Cell Bank (Ibaraki, Japan) [26,40,41,42]. MPM cell lines MSTO-211H (RRID: CVCL_1430) and JL-1 (RRID: CVCL_2080) were purchased from DSMZ (German Collection of Microorganisms and Cell Cultures, Brunswick, Germany). A primary MPM cell culture (BE261T) was established from surgically resected tumors of a 67 year-old male patient using the same protocol as described in [26,43] and used for short-term studies (up to eight passages in vitro). The human study was performed under the auspices of protocols approved by institutional review board (KEK number: 042/15), and informed consent was obtained from patients. Cells were cultured in RPMI-1640 medium or Medium 199 (Cat. #8758 and #4540; Sigma-Aldrich, St. Louis, MO, USA) supplemented with 10% fetal bovine serum/FBS (Cat. #10270-106; Life Technologies, Grand Island, NY, USA) and 1% penicillin/streptomycin solution (Cat. #P0781, Sigma-Aldrich, St.Louis, MO, USA). All human cell lines have been authenticated using STR profiling within the last three years and are confirmed free from mycoplasma contamination (Microsynth, Bern, Switzerland). Cisplatin, pemetrexed/MTA (Cat. #VL7640) and HA15 (Cat. #CS-5825) were obtained from Sandoz, Eli Lilly (Vernier, Suisse) S.A. (Vernier/Geneva, Switzerland) and ChemScene (Monmouth Junction, NJ, USA), respectively. 

Chemo-resistant cells (H28R, MESO-1R, and MESO-4R) were generated by chronic exposure to cisplatin/MTA following a weekly schedule of 4-day treatment and 3-day recovery. Drug treatment was initiated from cisplatin (0.1 µM) /MTA (0.5 µM) and increased in a stepwise manner until cisplatin (3 µM) /MTA (5 µM). Induction of resistance was measured by the cell viability assay. 

### 4.2. Cell Viability and Clonogenic Survival Assay

MPM cells seeded in 96-well plates (2500 cells/well) were dosed 24 h later with HA15 for 72 h. Cell viability was determined by acid phosphatase (APH) assay as described [26,43,44]. The efficacy of drugs on cell growth was normalized to the untreated control. Each data point was generated in triplicate and each experiment was done three times (*n* = 3). Unless otherwise stated, a representative result is presented. Best-fit curve was generated in GraphPad Prism [(log (inhibitor) vs. response (-variable slope four parameters)]. Error bars are mean ± s.d.

Clonogenic assay was performed as described [26,43,44]. In brief, exponentially grown MPM cells seeded in 6-well plates (1000 cells/well) were dosed 24 h later and continually treated with HA15 for 14 days (refresh drugs every three days), the resulting colonies were stained with crystal violet (0.5% dissolved in 25% methanol). Growth curve was generated by eluting crystal violet staining with 10% acetic acid and measuring absorbance at 590 nm. Three independent experiments were performed.

### 4.3. Quantitative Real-Time PCR (qRT-PCR) 

Total RNA was isolated and purified by RNeasy Mini Kit (Cat. #74106; Qiagen, Germany). Complementary DNA (cDNA) was synthesized by the high capacity cDNA reverse transcription kit (Cat. #4368814; Applied Biosystems, Foster City, CA, USA), according to the manufacturer’s instructions. Real-time PCR was performed in triplicate on a 7500 Fast Real-Time PCR System (Applied Biosystems) with commercially available TaqMan ‘Assay on Demand’ primer/probes: *EIF2AK3* (Hs00984005_m1), *ERN1* (Hs00980095_m1), *ATF4* (Hs00909569_g1), *DDIT3* (Hs00358796_g1), *BCL2* (Hs00608023_m1), *BCL2A1* (Hs00187845_m1), *BCL2L11* (Hs00708019_s1), *BBC3* (Hs00248075_m1), *ATG5* (Hs00355492_m1), *ATG7* (Hs00197348_m1), and *BECN1* (Hs00186838_m1). The expression of individual genes was normalized against *GAPDH* (Mm99999915_g1) using the ^ΔΔ^CT method. The baseline and threshold for CT calculation were set automatically with the 7500 software v2.06 (Thermo Fisher Scientific, Waltham, MA, USA).

### 4.4. Western Blot and Immunohistochemistry

Cell lysates were prepared and Western blot analysis was performed as described [26,43,44], with the exception that protease inhibitors (Cat. #78440; Thermo Fisher Scientific, Waltham, MA, USA) were included in lysis buffer. In brief, equal amounts of protein lysates (10–25 μg/lane) were resolved by SDS-PAGE (Cat. #4561033; Bio-Rad Laboratories, Hercules, CA, USA) and transferred onto nitrocellulose membranes (Cat. #170-4158; Bio-Rad). Membranes were then blocked in blocking buffer (Cat. #927-4000; Li-COR Biosciences, Bad Homburg, Germany) for 1 h at room temperature and incubated with appropriate primary antibodies overnight at 4 °C. BiP (Cat. #3183S), IRE1α (Cat. #3294S), PERK (Cat. #5683S), eIF2α (Cat. #5324S), Phospho-eIF2α (Ser 51) (Cat. #3398S), ATF4 (Cat. #11815S), Beclin-1 (Cat. #3495S), LC3B (Cat. #12741S), PARP (Cat. #9532S), Cleaved-caspase 7(Cat. #8438S) and Actin (Cat. #3700S) from CST (Cell Signaling Technology, Leiden, Netherlands). IRDye 680LT-conjugated goat anti-mouse IgG (Cat. #926-68020) and IRDye 800CW-conjugated goat anti-rabbit IgG (Cat. #926-32211) from Li-COR Biosciences were used at 1:5000 dilutions. Finally, signals of membrane-bound secondary antibodies were imaged using the Odyssey Infrared Imaging System (Li-COR Biosciences). 

Surgically removed xenograft tumors and liver sections were formalin-fixed and paraffin-embedded (FFPE) and stained with hematoxylin and eosin (H&E) using standard protocols. FFPE tumor blocks were sectioned at 4 μm, deparaffinized, rehydrated, and subsequently stained with an appropriate antibody (Caspase 3, Cat. #9664, CST) using the automated system BOND RX (Leica Biosystems, Newcastle, UK). Visualization was performed using the Bond Polymer Refine Detection kit (Leica Biosystems) as instructed by the manufacturer. Images were acquired using PANNORAMIC^®^ whole slide scanners and processed using Case Viewer (3DHISTECH Ltd., Budapest, Hungary).

### 4.5. Apoptosis Assays

MPM cells were treated for 72 h with vehicle control or HA15. After treatment, cells in the supernatant and adherent to plates were collected, washed with PBS, and pooled before suspended in 400 µL binding buffer and stained with the Annexin V Apoptosis Detection Kit-FITC (Cat. #88-8005; Thermo Fisher Scientific, Waltham, MA, USA) according to the manufacturer’s instructions. Flow cytometry analysis was performed on a BD Biosciences LSRII flow cytometer. Three independent experiments were performed.

### 4.6. siRNA Knockdown

Knockdown of CHOP was achieved by specific duplex siRNAs (50 nmol/L) purchased from Origene Technologies (Cat. #SR319903, Rockville, MD, USA). Transfection of siRNAs was performed with SiTran1.0 (Cat. #TT300001, Origene Technologies, Rockville, MD, USA), according to the manufacturer’s instructions.

### 4.7. In Vivo Mouse Study 

Mouse studies were conducted in accordance with Institutional Animal Care and Ethical Committee-approved animal guidelines and protocols. All mouse experiments were performed in age- and gender-matched NSG (NOD-*scid IL2Rγ^null^*). Suspensions of tumor cells (in PBS) mixed with 1:1 with BD Matrigel Basement Membrane Matrix (Cat. #356231; Corning Inc., Corning, NY, USA) were subcutaneously inoculated in left and right flanks (BE261T cells: 1 × 10^6^/injection). When tumors were palpable, mice were randomly assigned to treatment groups: (1) control; (2) cisplatin (3.75 mg/kg) plus pemetrexed (83 mg/kg) (i.p., once weekly) for five weeks; (3) HA15 (0.7 mg/mouse, i.p., 5 days/week) for five weeks. Mice weight and tumor size were measured every three days. Tumor size was calculated as follows: (length × width × width)/2. At the endpoint, blood samples were collected through cardiac puncta and subjected to the determination of transaminase activities. 

### 4.8. Public Databases (GEO, GSEA, TCGA, EMBL-EBI) and UPR Gene Signature 

Transcriptomic dataset of normal and MPM tumor samples (GSE2549 and GSE51024) were downloaded from the Gene Expression Omnibus (GEO) database [31,32] and subjected to gene set enrichment analysis (GSEA). Transcriptomic data of cancer patients were obtained from the Cancer Genome Atlas (TCGA) (https://portal.gdc.cancer.gov/projects/TCGA). The expression of key UPR genes in MPM cell lines were downloaded from the European Bioinformatics Institute of European Molecular Biology Laboratory (EMBL-EBI, http://www.ebi.ac.uk/). The UPR status of tumors was determined by the UPR gene signature, scored as the sum of the reactome unfolded protein response gene set.

### 4.9. Statistical Analysis

Statistical analyses were performed using GraphPad Prism 7.01 (GraphPad Software Inc., San Diego, CA, USA) unless otherwise indicated. All samples that met proper experimental conditions were included in the analysis and sample size was not pre-determined by statistical methods, but rather based on preliminary experiments. Group allocation was performed randomly. In all studies, data represent biological replicates (*n*) and are depicted as mean values ± s.d. or mean values ± SEM as indicated in the figure legends. Comparison of mean values was conducted with unpaired, two-tailed Student’s *t*-test, one-way ANOVA, or two-way ANOVA with Tukey’s multiple comparisons test as indicated in the figure legends. In all analyses, *P* values less than 0.05 were considered statistically significant. Gene expression and survival data derived from the public database as well as the correlation coefficient were analyzed using R (Version 3.4.3, https://cran.r-project.org/). For the survival analysis, patients were grouped by gene expression, where ‘high’ and ‘low’ expression groups were stratified by the optimal cut-off value.

## 5. Conclusions

Our study revealed that dysregulation of ER stress and adaptive UPR pathway is a characteristic feature of MPM, and supports further investigation of HA15 as a novel therapeutic for patients with MPM. 

## Supplementary Materials

The following are available online at https://www.mdpi.com/2072-6694/11/10/1502/s1, Figure S1: Uncropped blots from Figure 1F, Figure 3C−E, Figure 4C,G, Figure 5G and Figure 6G. Numbers next to the blot indicate molecular weight (kDa) of the marker.
