# Supplementary material for: Endoplasmic Reticulum Stress Signaling as a Therapeutic Target in Malignant Pleural Mesothelioma
Source: Cancers (Basel). 2019 Oct 8;11(10):1502. doi: 10.3390/cancers11101502 (PMC6827154; doi:10.3390/cancers11101502)
Supplement: Supplementary file 1 [file cancers-11-01502-s001.pdf]

Supplementary Materials: Endoplasmic reticulum stress signaling as a therapeutic target in malignant pleural mesothelioma

A

Figure 1F

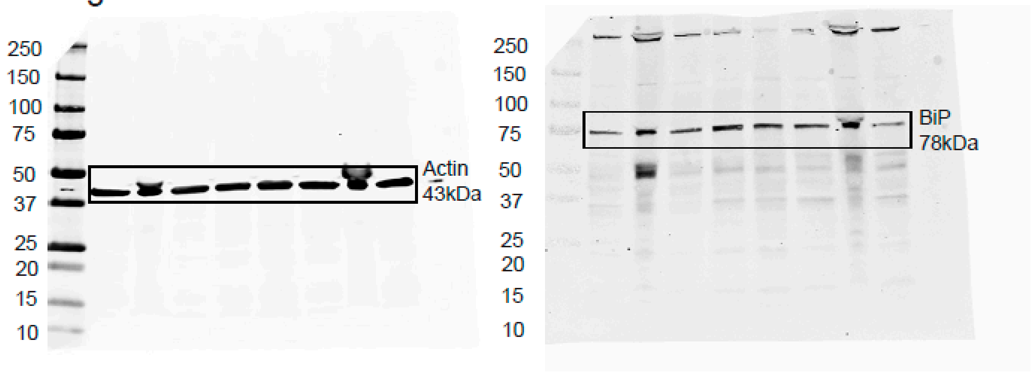

B

Figure 3C

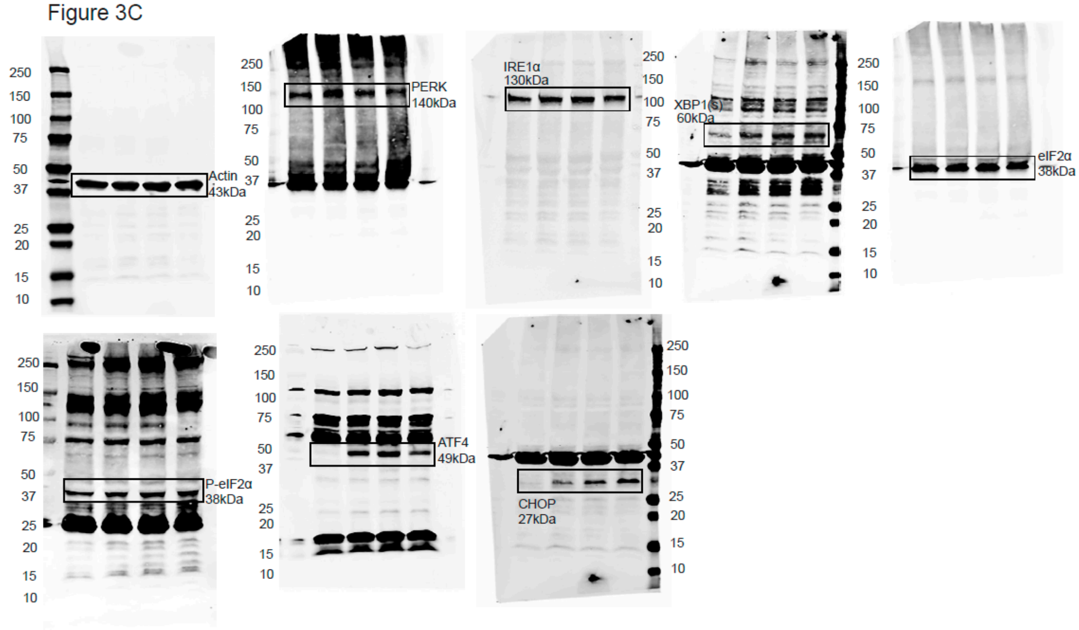

C

Figure 3D

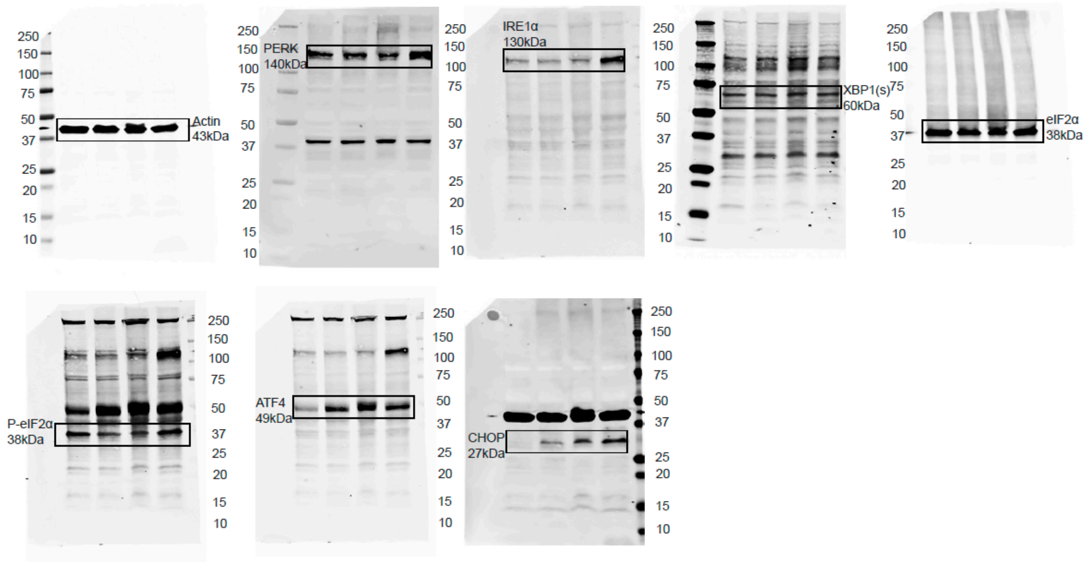

D

Figure 3E

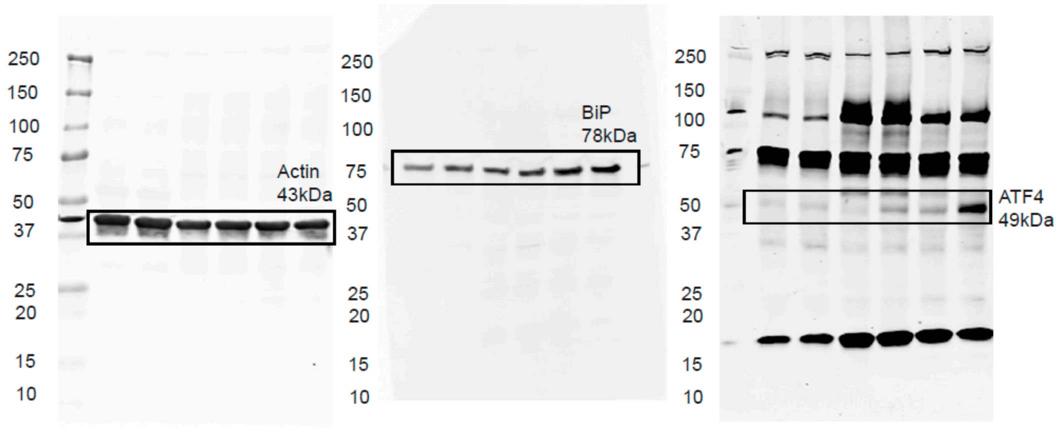

E

Figure 4C

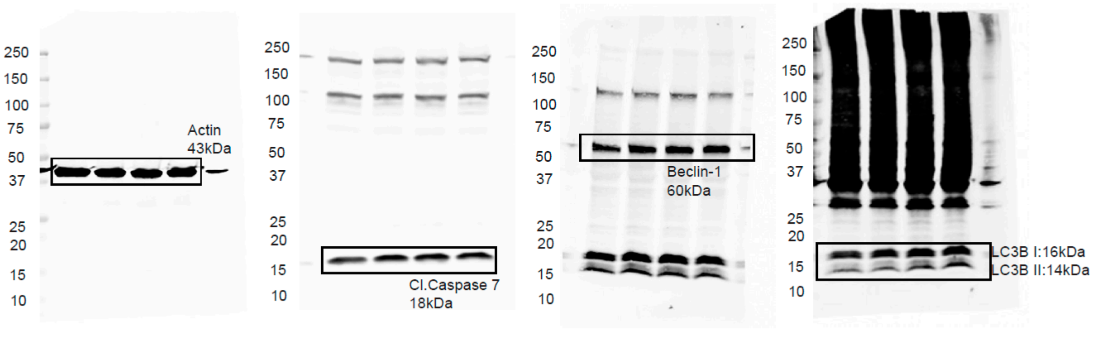

F

Figure 4G

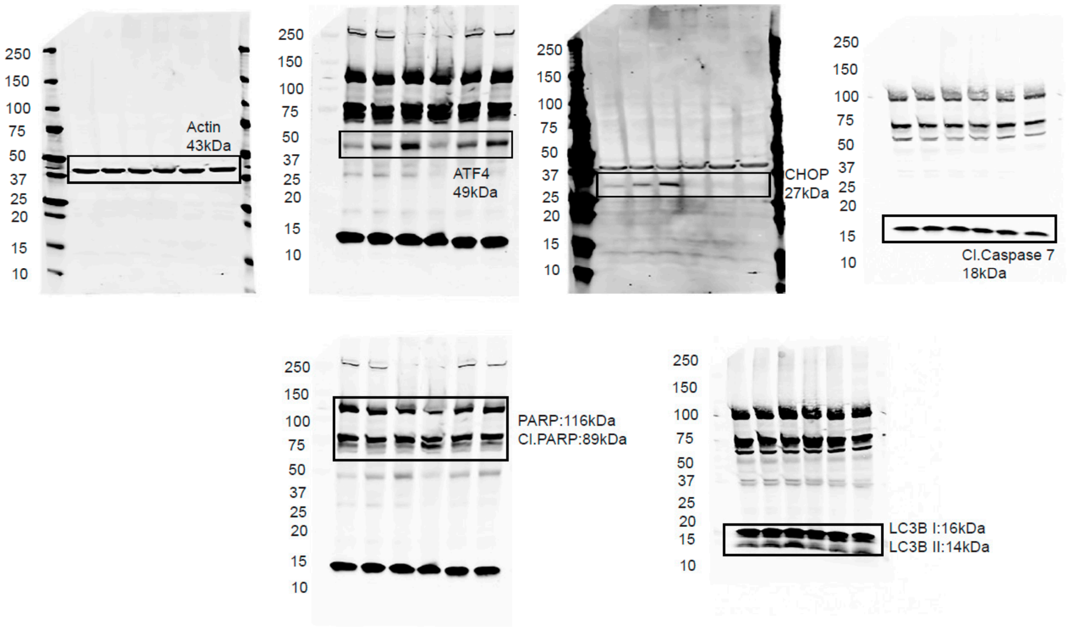

G

Figure 5G

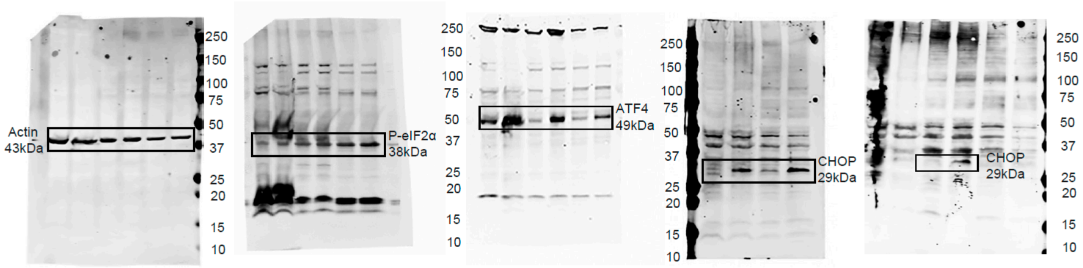

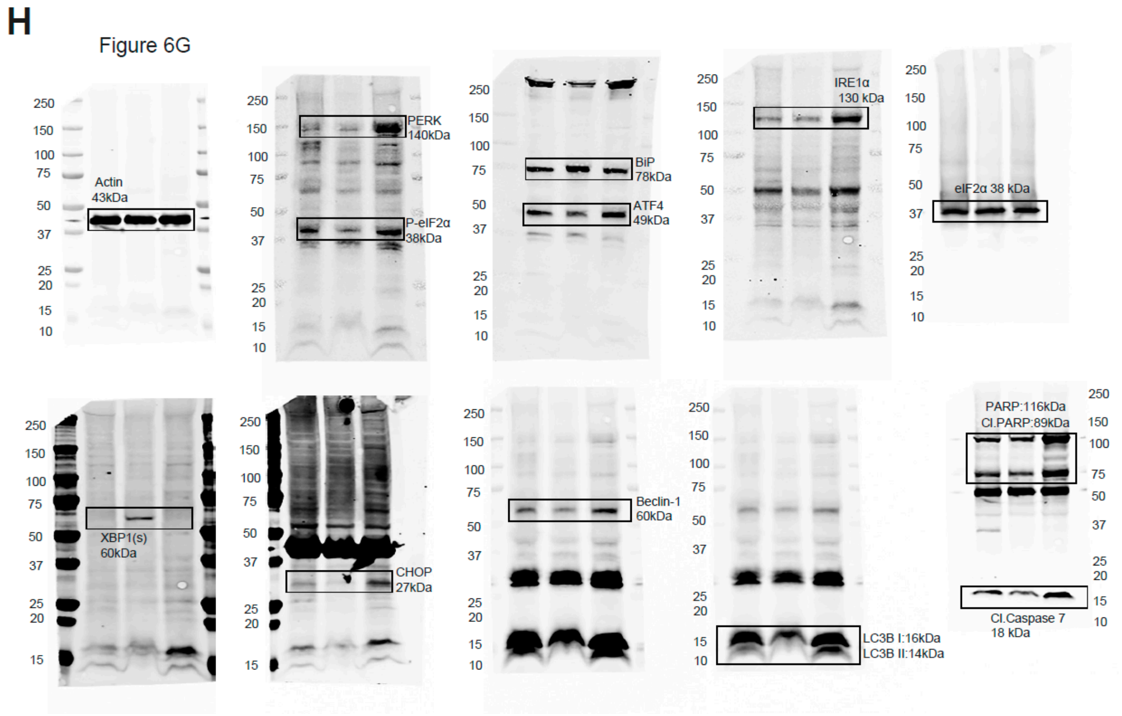

Figure S1 (A-H). Uncropped blots from Figure 1F, 3C-E,4C, G, 5G and 6G. Numbers next to the blot indicate molecular weight (kDa) of the marker.
